# Beam Deflection Monitoring Based on a Genetic Algorithm Using Lidar Data

**DOI:** 10.3390/s20072144

**Published:** 2020-04-10

**Authors:** Michael Bekele Maru, Donghwan Lee, Gichun Cha, Seunghee Park

**Affiliations:** 1 School of Civil, Architectural Engineering and Landscape Architecture, Sungkyunkwan University, Suwon 16419, Korea; mikemaru@skku.edu; 2 Department of Convergence Engineering for Future City, Sungkyunkwan University, Suwon 16419, Korea; ycleedh@gmail.com (D.L.); ckckicun@naver.com (G.C.)

**Keywords:** terrestrial laser scanning (TLS), point cloud, deflection, genetic algorithm (GA), curve fitting

## Abstract

The Light Detection And Ranging (LiDAR) system has become a prominent tool in structural health monitoring. Among such systems, Terrestrial Laser Scanning (TLS) is a potential technology for the acquisition of three-dimensional (3D) information to assess structural health conditions. This paper enhances the application of TLS to damage detection and shape change analysis for structural element specimens. Specifically, estimating the deflection of a structural element with the aid of a Lidar system is introduced in this study. The proposed approach was validated by an indoor experiment by inducing artificial deflection on a simply supported beam. A robust genetic algorithm method is utilized to enhance the accuracy level of measuring deflection using lidar data. The proposed research primarily covers robust optimization of a genetic algorithm control parameter using the Taguchi experiment design. Once the acquired data is defined in terms of plane, which has minimum error, using a genetic algorithm and the deflection of the specimen can be extracted from the shape change analysis.

## 1. Introduction

In the 4th industrial revolution, Structural Health Monitoring(SHM) is becoming a hot issue in the construction industry [1,2] in which nondestructive evaluation and serviceability monitoring are essential features. Nondestructive evaluation plays a critical role in assuring that structural components and systems perform in a reliable and cost-effective fashion. This mechanism does not affect the future usefulness of the object or material. On the other hand, serviceability monitoring is performed in real time throughout its service life span [3,4]; this is directly related to controlling the structural responses caused by either deflection, cracks, vibration, creep, or a combination of them. Various techniques have been utilized for the assessment of structure performance [5]. Sensors are essential components and have different purposes based on the nature of the techniques. In general, we can classify sensors into two types based on the connection. Contact sensors which are commonly known for having physical interactions with the target structure. A Linear Variable Differential Transformer(LVDT), piezoelectric transducer, fiber optic sensor and acoustic emission sensor are common examples of this type [6,7,8]. On the other hand, non-contact sensors are known for acquiring responses from the target material without making direct or indirect contact. Laser sensor systems, drones with vision-based sensors using cameras, wireless rechargeable sensor networks, radar sensor networks and lidar sensor systems are grouped as this type [9,10,11]. Recently, non-contact sensors are being commonly utilized due to their portability, easy use in harsh surroundings, and so on. Knowing about the deflection of a beam or any structure using TLS has a big advantage from the perspective of site conditions. For the sake of describing the benefits of this study in the real world, aged structures, considering their spatial positions and site conditions, are not safe due to perilous structural conditions, inconvenience, insecurities and slippery site conditions. Consequently, the Light Detection And Ranging (LiDAR) system has become prominent in structural health monitoring. Despite such sensors being used for detection, measurement and characterization of hidden and/or apparent defects using advanced techniques, there are still many questions regarding the dimensions of the sensors for structural health monitoring.

Terrestrial Laser Scanning (TLS), or lidar, is a crucial non-contact optical sensor that analyzes the structural Three Dimensional(3D) shape in terms of a very dense 3D point cloud. This technology is a recent innovation in the spatial information of data acquisition, allowing for a scanned area to be digitally captured with unprecedented resolution and precision. However, the acquired data are influenced by several factors. Among them, random errors due to inherent physical properties are often difficult to eliminate while error due to environmental and scanning geometry issues can be removed through analysis [12]. Consequently, attention should be given to data acquisition by considering the position, incidence angle, and color of a specimen [13]. As a result, it is possible to dwindle noises tremendously.

In recent years, various approaches have been used to estimate the elements for serviceability limit states such as accumulated deflection, crack and dynamic displacement [14,15,16]. Several researchers have published papers concerning the serviceability assessment in support of structural health monitoring [17,18,19]. Park et al. conducted an experiment to define the deflection of a steel structure element via TLS using the geometrical shape of the specimen [20]. Even though the model of Cabaleiro et al. is not valid due to torsion, they tried to model a deformed beam caused by concentric loads and torsional forces on the specimen after scrutinizing the max deflection with respect to the allowable building codes [21]. Some researchers proposed how to estimate the deflection of a structural element, i.e., a beam, by integrating photogrammetry with TLS. For instance, Gordon measured the beam deflection with respect to benchmark photogrammetric data [22]. Zogg and Ingensand tried to monitor a deformation of real structure called Felsenau Viaduct(CH) bridge which is a part of Swiss highway via with TLS. The deformation on their study is mainly caused by settlement and tilting of a structure. Eventually, they showed that TLS could replace the area wide precise levelling in monitoring of a structure deformation since the maximum difference in between these two system is less than 1 mm [23]. Olsen et al. conducted a damage assessment for full scale structural test specimen by identifying volumetric change and its deformation [24]. Cabaleiro et al. utilized TLS data for checking of a deflection and stresses caused by torsion especially for open cross section which have a very low torsional strength. They compared the result obtained from the proposed methodology with the measurement taken during an experiment and Finite Element Modeling(FEM). As a result, they could show that their proposed algorithm is much closer to the measurement taken directly which is considered as a ground truth data [25].

Naturally, Lidar data are highly vulnerable and easily affected by noise [26]. Different scholars have been studying the factors which affect the Lidar data regarding structural health monitoring [13,27]. On the other hand, some researchers have used different types of denoising methods and optimization techniques in order to diminish their effect [28,29]. Notice that, outliers and noise are differing conceptually. Indeed, these two terms have really ambiguous meaning to differentiate by the researchers. However, Sagado et al. defined outliers and noise individually. According to their definition, an outlier is a data point which is different from the remaining data whereas noise can be defined as mislabeled examples (class noise) or errors in the values of attributes (attribute noise) [30]. Genetic Algorithm(GA) is also one of the crucial optimization methods used in order to attain the target effectively without the noise affection. GA has been conducted in different topics of structural health monitoring by different scholars. Optimization of sensor placement which provides the best possible performance is one of many critical topics [31]. However, estimating a structural deflection for the purpose of assessing the performance via a genetic algorithm is not prominently conducted so far. Rather, many researchers have performed this optimization method to determine a structure deflection both analytically and numerically [32]. GA can be also incorporated with a regression methodology to increase the robustness of curve fitting. Indeed, there are many techniques that are capable to increase the robustness of a curve fitting technique when we have numerous numbers of outliers in the data; Least Absolute Deviation (LAD), M-estimation, and S estimation are popular schemes which can considerably improve estimation precision [33].

This study presents an effective algorithm for measuring the structural deflection by enriching previous studies. Improvements are made according to the following five main steps of the procedure listed; the acquisition of TLS data for the loading and unloading scenarios, fitting of a plane for the point cloud acquired during the unloading scenario using a robust genetic algorithm, transformation of the scanner coordinates into local structural coordinates, curve fitting of transformed data for the loading case, and eventually, estimation and comparison of deflection between contact sensors. Furthermore, our research illustrates the performance of the proposed procedure with a validating experiment in which deflection measurements are simulated based on the loading scenario.

## 2. Basic Principles

### 2.1. Least Square Regression

A plane can be described by a normal vector $n={\left[A,B,D\right]}^{T}$ perpendicular to the plane, and a vector on the plane connected to a known point $p_{1}$ = ($x_{1}$, $y_{1}$, $z_{1}$) and an arbitrary point $p_{2}$ = (*x*, *y*, *z*) is described by $p_{2}-p_{1}$ since the normal vector and vector in the plane are perpendicular to each other, the dot product of these two vectors should be null [34].

(1) $$\vec{n}\cdot \left(\vec{P_{2}}-\vec{P_{1}}\right)=0\;\;\;∵\;\;\;\left(\vec{n}⊥\left(\vec{P_{2}}-\vec{P_{1}}\right)\right)$$

For the sake of simplifying the over-determined problem, begin by removing one component by constraining the solution space. Thus we assume the coefficient D is the unit:

(2) $$Ax_{i}+By_{i}+z_{i}+C=0$$

Keeping the assumption that the *z*-component of the data is functionally dependent on the *x* and *y*-components and given a set of samples $\left(x_{i},y_{i},z_{i}\right)$, determine *A*, *B*, and *C* so that the plane *z* = *Ax* + *By* + *C* best fits the samples in the sense that the sum of the squared errors between the $z_{i}$ and the plane values $Ax_{i}+By_{i}+C$ is minimized [35]. This can be written via Equation (3).

(3) $$E\left(A,B,C\right)={\sum \left[z_{i}-\left(Ax_{i}+By_{i}+C\right)\right]}^{2}$$

### 2.2. Genetic Algorithm

Stochastic optimization is a process of seeking a maximum or minimum value of a mathematical or statistical function in the presence of randomness. GA is one of the common method which include in this kind of optimization techniques. It is used to find the optimal solution(s) to a given computational problem maximizing or minimizing a function. A genetic algorithm is a random-based classical evolutionary algorithm [36]. This principle of continual improvement over generations is utilized by evolutionary algorithms to optimize solutions to a problem. In the initial generation, a population composed of different individuals is generated randomly or by other methods. An individual is a solution to the problem, which can vary in quality: the quality of an individual with regard to the problem is called the fitness, which reflects the adequacy of the solution to the problem to be solved. The higher the fitness of an individual, the more likely it is to pass some or all of its genotype to individuals of the next generation. Increasing the robustness of an algorithm improves the output. The primary distinguishing features of such algorithms are encoding, a selection mechanism, a crossover mechanism, a mutation mechanism, and a culling mechanism. Such algorithms can optimize multiple objectives simultaneously and can be used as black boxes since they do not assume any properties of the mathematical model to be optimized [37]; their only real limitation is the computational complexity.

## 3. Proposed Approach for Computation of Deflection

**Figure 1:**
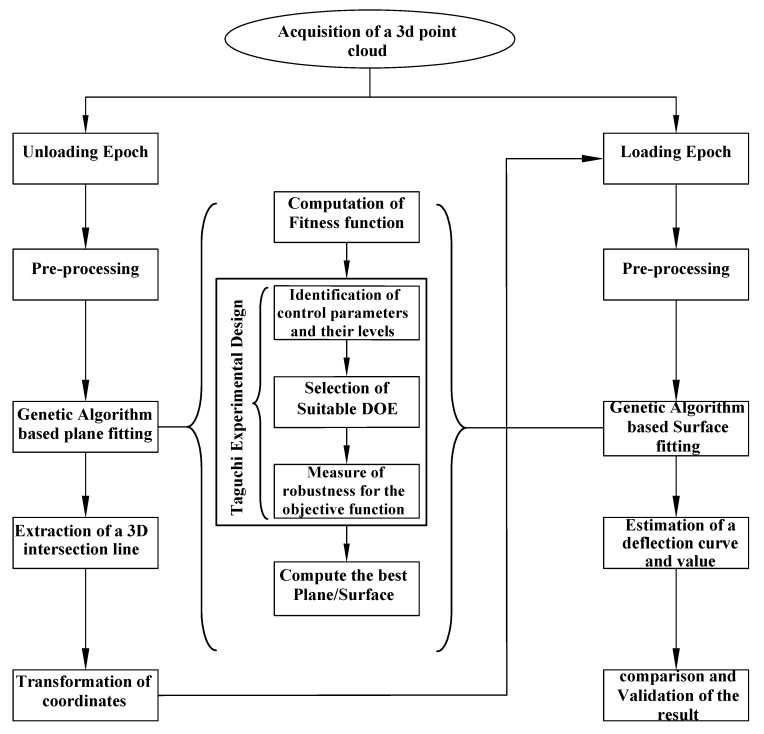
Flow chart for the proposed method.

The proposed method is applied to measure artificial deflection of a structural element from TLS data and compare this with the contact sensor mounted during the experiment. The deflection is estimated by taking the difference in shape change between the loading and unloading scenarios. In the unloading case, first the resulting point cloud data are represented by plane and an extracted line, which help to set the spatial position of a local specimen coordinate system. Once the coordinate system is transferred to the new system, we can define and visualize a deflection curve for each loading case. The flowchart in Figure 1 shows the steps needed to estimate the deflection of a structure. The subsequent subsections explain the proposed method in detail.

### 3.1. Acquisition of the Point Cloud and Pre-Processing

**Figure 2:**
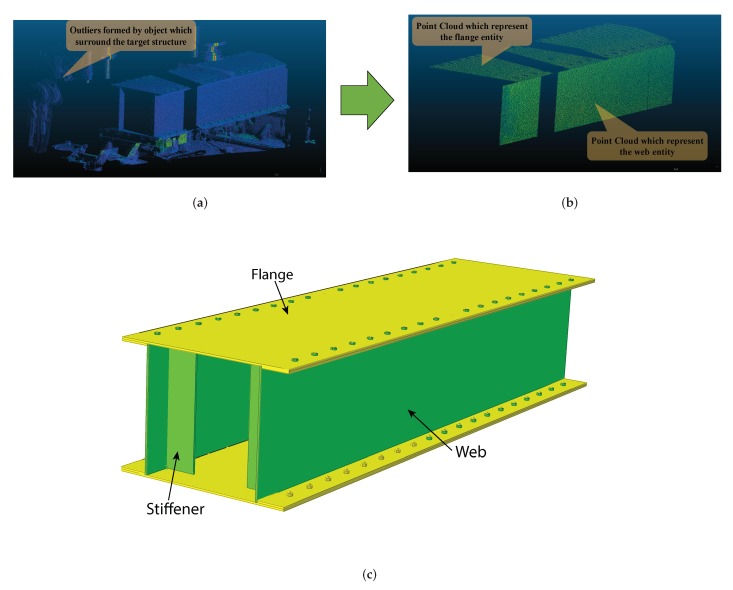
(**a**) Point cloud before preprocessing; (**b**) Point cloud after preprocessing; (**c**) solid figure of a specimen.

Representing or modeling real-world scenarios with virtual worlds makes it easy to apply analytical theories and visualize the effects. Among them, capturing an object using a 3D digital device, i.e., TLS in our case, enables us to measure accurately any changes in shape with magnificent resolution. Basically, The TLS point cloud tells us the spatial position (*X*,*Y*,*Z*) of a point with respect to its own scanner coordinate system. Additionally, TLS provides us with information about the scalar field that represents intensity, RGB color information, and time in some cases. Indeed, not every sensors can produce RGB color information for the users. Some brands like, RIEGL LMS-Z420i, has a camera system that detached from the main system which is used to acquired the color data of an objects. whereas in our case the system has in-built camera system which helps to produce RGB color data with the corresponding *X*, *Y*, and *Z* coordinates. In this study, only the spatial information of a point cloud is utilized for the proposed analysis to achieve the required solution. Pre-processing is a technique that improves the accuracy and resolution of data, along with filtering techniques that serve to enhance or highlight the spatial characteristics of an image data set. Manual trimming and segmentation are employed in this study since they are considered enough for analyzing the data. After removing the outliers and segmentation, the number of points on the flange became 39,730 while those on the web entity become 163,024 points. Figure 2 depicts the resulting scanned point cloud representation of a specimen with the corresponding solid 3D representation of an object.

### 3.2. Characterizing the Point Cloud in Terms of Definable Mathematical Elements

Our data form a point cloud set which has information about its spatial position. We only have a collection of point cloud data which describe the specimen. Therefore, representing this data by a plane and/or a surface, which can be defined by some mathematical equation, is significant for further analysis. Extracting the line which helps us to locate the local structural coordinate system, transforming the coordinate of a point from scanner coordinate system to local structural coordinate system and computing the deflection of a specimen anywhere along with the span are some tasks that can be computed whenever the acquired point cloud data is defined by mathematical plane and/or surface.

#### 3.2.1. Least Squares Genetic Algorithm(LS-GA) Based Plane Fitting

**Figure 3:**
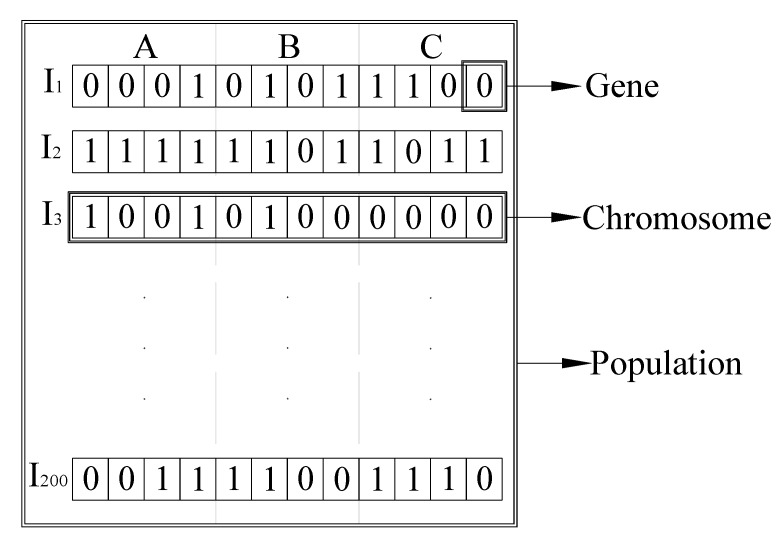
GA Basic Features.

The principle of least squares sum in the curve fitting problem, which minimizes the difference between the data and model of the output, is useful. However, in this study, the basic principle of least squares is incorporated with a notable stochastic optimization known as a genetic algorithm for the purpose of decreasing the difference between the data and model [38]. Consequently, this is particularly useful if we utilize an error metric in terms of the functional form and optimize it using a GA. Holland and his student stated that GA works with an encoding of the individuals throughout the algorithm [36]. Originally, they constructed a GA based on binary numbers. Once we have fixed the number of chromosomes in the population, the basic algorithm operators (Selection, Crossover, and Mutation) start to conduct iteratively until it converges to optimal solutions. Currently, all these complication and processes are simplified using different platforms. Among them, the global optimization toolbox in MATLAB software is utilized for our studies. The essential parameters for running the GA in this study are [37]:

- Fitness function: The fitness function is a mathematical formulation of the desired optimization problem. It determines how suitable a solution is. The magnitude of the residual, which defines the difference between the model and data examines the individuals formed throughout all generation. This can be described in Equation (4):
  (4) $$Fitness\;value=\sum_{i=0}^{N}{d_{i}}^{2}=\sum_{i=0}^{N}{\left(P_{i}-F\left(A,B,C\right)\right)}^{2}$$
  where F(A,B,C) is the point model, normally defined as the nearest data point *P*
- Population: The population is a set of individuals that have a chance to be the fittest among them. An individual is characterized by a set of variables called its genes. It is a basic building block of for the algorithm. In our case, this refers to the parameter of the regression model. These genes are joined into a string to form a chromosome (individuals). It is simply a series of binary numbers (0 and 1), which encode all values of parameter of the regression model. $I_{1},I_{2}$ up to $I_{200}$ that are shown in Figure 3 are list of chromosomes in the population which have a binary representation of all the parameters *A*, *B*, and *C* of the plane. Our GA is one of the unique behaviors of this optimization techniques; it works only on the number of chromosomes inside the population. The number of chromosomes in the population defined by the user is labeled as the population size. The building block and their corresponding chromosomes in the population is shown in the Figure 3.
- Fitness scaling function: If we consider only multiplying the fitness by some factor, then as the name implies, this does not change the relationships among the population. Rather, a much more sensible fitness scaling is an affine transformation, where scaled(f) = a ∗ f + b, of the fitness value, is seen via formulae, the values are multiplied by some number and then offset by another either up or down. This parameter plays a crucial role in GA by limiting the tendency of the strongest solution to overwhelm the weaker ones thus avoiding premature convergence.
- Selection: The selection is a parameter for choosing two parents from the population for the purpose of mating. This process continues in every single generation until one of the stopping criteria is attained. Parents, i.e., a pair of individuals, are chosen based on their fitness score.
- Crossover/Reproduction: This parameter mainly depicts how the selected individuals mate with each other and create new offspring for the next generation. In this process, part of the individuals is exchanged with its mate based on the user-defined crossover probability.
- Elite Count: This is the number of individuals with the best fitness values in the current generation that have been retained for the next generation. Because of their robust fitness score, more copies of these individuals are achieved in the subsequent generation.
- Crossover fraction: The fraction of individuals in the next generation, other than the elite children, that are created via crossover.
- Mutation: A process of changing of a bit (gen) within a bit string (chromosome). This is done to maintain diversity within the population and prevent premature convergence.
- Migration: An exchange of information (exchange of individuals) between the sub-populations.

**Figure 4:**
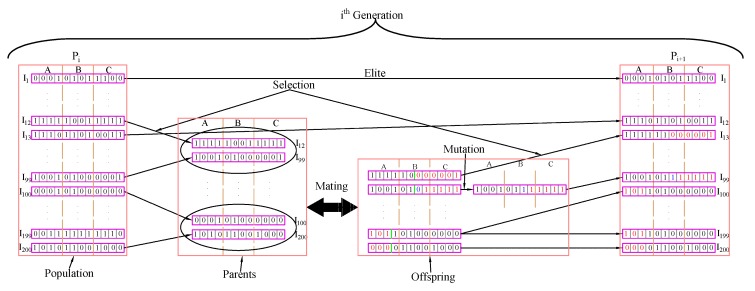
Genetic Algorithm Setup.

Figure 4 illustrates how the parameter of genetic algorithm is functioning in one generation. This feature of a genetic algorithm allows us to find the optimal parameter of a plane with respect to an error metric.

#### 3.2.2. Taguchi Experimental Design

Even though the prevalent method of modeling a plane fitting is based on deterministic ordinary differential equations, a stochastic method such as a genetic algorithm is more appropriate and well established whenever the amount of noise is significant [38,39,40]. However, GAs have limitations regarding the optimality of individuals which satisfy the given fitness function unless an appropriate input parameter is selected. Consequently, to overcome this drawback, a different scheme has been utilized for tuning a suitable parameter for a genetic algorithm [41,42,43]. Among various techniques, the Taguchi experimental design approach is used for tuning the parameters of the GA solver. Dr. Taguchi developed the design of an experiment based on well-defined guidelines. Taguchi’s design uses orthogonal arrays that estimate the effects of factors on the response mean and variation. An orthogonal array means the design is balanced such that the factor levels are weighted equally. Taguchi uses the following convention for naming the orthogonal arrays: $L_{a}\left(b^{c}\right)$, where *L* stands for Latin square, *a* is the number of experimental runs, *b* is the number of levels of each factor, and c is the number of variables [44]. We can easily assess each factor individually because of utilizing an Orthogonal Array on the design experiment. As a result, the effect of one factor does not affect the estimation of a different factor. This can reduce the time and cost associated with the experiment when fractionated designs are used. Therefore, Taguchi’s design of experiments plays a crucial role in the reduction of number of trial experiments [45]. The parameters in Taguchi’s design are selected by considering the effects of individual value on the algorithm output. Here, nine parameters and their levels are selected and summarized in Table 1.

Among the various preferences for experiment design, a robust design is identified using the signal-to-noise ratio (SNR). This is because the higher values SNR identify the control settings that minimize the effects of the noise factors. Basically, the Taguchi design of experiment is used for two optimization processes: (a) use the signal-to-noise ratio to identify those control factors that reduce variability. (b) identify control factors that shift the mean to the target and have a small or no effect on the signal-to-noise ratio [45]. A SNR is suggested by Taguchi for cases in which the response standard deviation is related to the mean. The purpose of SNR in Taguchi’s approach to robust parameter design is to provide an easy-to-use performance criterion that takes the process mean and variance into account. Among the numerous SNR values, “the smaller-the-better” philosophy used in this study, as shown in Equation (5).

(5) $$SNR=-10log\left[\sum_{i=1}^{n}\left(\frac{{y_{i}}^{2}}{n}\right)\right]$$

where; SNR is signal to noise ratio, $y_{i}$ is the response variables, and *n* is number of response values. $\sum_{i=1}^{n}$ implies the summation over n response values at the outer array points.

#### 3.2.3. Extraction of the 3D Intersection Line

**Figure 5:**
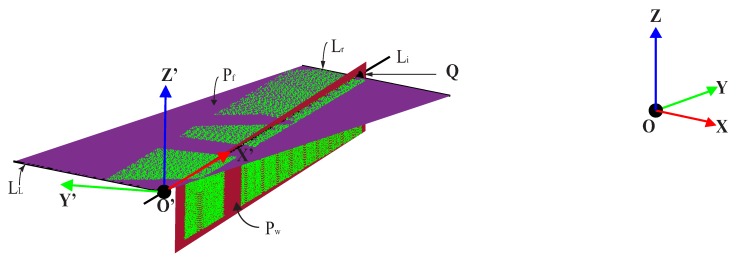
Plane representation for web and flange part.

Defining the coordinates of points using the new basis is important because it allows for visualization and quantification of the real shape changes of the specimen. However, it is not easy to find out the appropriate position of a new basis (local structural coordinate system) since we have a collection of points. Therefore, considering one axis (in this case, the X-axis) coincides with intersection 3D line between the flange fitted plane $P_{f}$ and the web fitted plane $P_{w}$ is an efficient way of determining the position with the new basis as shown in the Figure 5. Suppose the direction vectors for the flange and web planes in the Figure 5 are $N_{f}\left(i_{f},j_{f},k_{f}\right)$ and $N_{w}\left(i_{w},j_{w},k_{w}\right)$, respectively. Since these two planes cross each other perpendicularly, the vector product of their normal vectors is equivalent to the direction vector *S* of their line of intersection, $L_{i}$ (6).

(6) $$N_{f}\left(i_{f},j_{f},k_{f}\right)\times N_{w}\left(i_{w},j_{w},k_{w}\right)=S$$

However, this information is not enough to extract the equation of the line. Thus, picking one arbitrary known point for our case results in the intersection of the line of intersection of the two planes $L_{i}$ with the left edge of the plane $L_{l}$, and this point is inserted in to the following symmetric form in Equation (7) for 3D lines.

(7) $$\frac{x-x_{0}}{a}=\;\frac{y-y_{0}}{b}=\frac{z-z_{0}}{c}=t$$

where; *a*, *b* and *c* are direction vectors of the line $L_{i}$ i.e., S=(a,b,c); $x_{0}$, $y_{0}$ and $z_{0}$ are the coordinates of a point on a line, taken here as (0,−7.495,−0.402).

Accordingly, the real spatial position of the new basis (*x*′,*y*′,*z*′) can be used to determine the coordinate transformation, followed by setting the x′-axis of the local structural coordinates on the extracted intersection line of the planes, as shown in Figure 5.

#### 3.2.4. Transformation of Coordinates

To easily evaluate the real changes in shape of the specimen by visualizing the effect of loading, the reference frame is transformed from the scanner coordinate system to the local structural coordinate system. This implies that the acquired data set coordinates should be defined using the local structural coordinate system. A rigid transformation (also called an isometry) is a transformation which preserves the shape and size of the object. Reflections, translations, rotations, and/or combinations of these three transformations can be categorized as rigid transformations [46]. A translation $T_{O^{\prime }O}$ is a transformation which slides an object by a fixed range from point *O* to point *O*′, as in Figure 5. All the other points move the same distance in the same direction whenever the coordinates of the point cloud are multiplied by the translation matrix, as shown by Equation (8). After a translation, which is defined as moving the origin of the scanner coordinates to the hypothetical spatial position of the local structural coordinates, a rotation is applied. A rotation $R_{\theta \_i,O\prime }$ is a transformation that twirls an object about a fixed point *O*′, called the center of rotation, with an anlge θ in either the *x*, *y*, *z*, or a combination of them; the rotation directions are called the Euler angles [47].

(8) $$\left[T\right]=\left[\begin{array}{ccc} 1 & 0 & \;\;\;\begin{array}{cc} 0 & \;\;0 \end{array}\\ 0 & 1 & \;\begin{array}{cc} \;\;0 & \;\;0 \end{array}\\ \begin{array}{c} 0\\ X_{O}^{\prime } \end{array} & \begin{array}{c} 0\\ Y_{O}^{\prime } \end{array} & \begin{array}{cc} \begin{array}{c} \;\;1\\ {\;\;Z}_{O}^{\prime } \end{array} & \begin{array}{c} \;0\\ 1 \end{array} \end{array} \end{array}\right]$$

The rotation matrices for rotating a vector about the x-axis by an angle α, about y-axis by an angle β and about the z-axis by an angle γ are given by Equations (9a)–(9c), respectively.

(9a) $$R_{\left(\alpha ,\;\;x^{\prime }\right)}\;=\;\left[\begin{array}{ccc} 1 & 0 & \;\;\;\begin{array}{cc} 0 & \;\;\;\;\;\;\;\;0 \end{array}\\ 0 & \cos\alpha & \;\begin{array}{cc} -\sin\alpha & \;\;0 \end{array}\\ \begin{array}{c} 0\\ 0 \end{array} & \begin{array}{c} \sin\alpha \\ 0 \end{array} & \begin{array}{cc} \begin{array}{c} \;\;\cos\alpha \\ 0 \end{array} & \;\begin{array}{c} 0\\ 1 \end{array} \end{array} \end{array}\right]$$

(9b) $$R_{\left(\alpha ,\;\;y^{\prime }\right)}\;=\;\left[\begin{array}{ccc} \cos\beta & 0 & \begin{array}{cc} \sin\beta & 0 \end{array}\\ 0 & 1 & \;\begin{array}{cc} \;\;0 & \;\;\;\;0 \end{array}\\ \begin{array}{c} -\sin\beta \\ 0 \end{array} & \begin{array}{c} 0\\ 0 \end{array} & \begin{array}{cc} \begin{array}{c} \cos\beta \\ 0 \end{array} & \begin{array}{c} 0\\ 1 \end{array} \end{array} \end{array}\right]$$

(9c) $$R_{\left(\alpha ,\;\;z^{\prime }\right)}\;=\;\left[\begin{array}{ccc} \cos\gamma & -\sin\gamma & \begin{array}{cc} 0 & 0 \end{array}\\ \sin\gamma & \cos\gamma & \begin{array}{cc} 0 & 0 \end{array}\\ \begin{array}{c} 0\\ 0 \end{array} & \begin{array}{c} 0\\ 0 \end{array} & \begin{array}{cc} \begin{array}{c} 1\\ 0 \end{array} & \;\begin{array}{c} 0\\ 1 \end{array} \end{array} \end{array}\right]$$

Once the transformation of a rotation angle into a rotation matrix is successfully completed, the transformation matrix in Equation (10) is obtained by combining the above-described rotation matrices with the translation matrix as follows:

(10) $$\left[M\right]=\;\left[T\right]\cdot \left[R_{\left(\alpha ,{\;x}^{\prime }\right)}\right]\cdot \left[R_{\left(\beta ,{\;y}^{\prime }\right)}\right]\cdot \left[R_{\left(\gamma ,{\;z}^{\prime }\right)}\right]$$

where, [M] is 4 × 4 transformation matrix; $\left[R_{\left(\alpha ,{\;x}^{\prime }\right)}\right]$ is a rotation matrix for rotating of a vector by an angle α with respect to the *x*′ axis; $\left[R_{\left(\beta ,{\;y}^{\prime }\right)}\right]$ is Rotation matrix for rotation of a vector by angle β with respect to *y*′ axis; and $\left[R_{\left(\gamma ,{\;z}^{\prime }\right)}\right]$ is Rotation matrix for rotation of a vector by angle γ with respect to *z*′ axis.

In the three-dimensional world, four coordinates are necessary when considering the perspective of a scene. In projective space, two parallel lines appear to meet at the horizon, which is not the case in Euclidean space. Therefore, mathematicians use homogeneous coordinates which can represent the N-dimensional coordinates using N + 1 numbers [48]. Equation (11) shows, how Cartesian coordinates can be described in terms of homogeneous coordinates. Consequently, the transformation matrix [*M*] is defined as a 4 × 4 matrix by redefining the rotation and translation matrices in terms of adding an additional dimension to the coordinates.

(11) $$\left(X,Y,Z\right)\equiv \left(x,y,z,w\right)\;\;\;\;\forall \;\;\;\;\{\begin{array}{l} X=x/w\\ Y=y/w\\ Z=z/w \end{array}$$

where, (X,Y,Z) are the Cartesian coordinates and (x,y,z,w) are the homogeneous coordinates.

Now, the coordinates of a point cloud acquired in a different loading are multiplied by the transformation matrix described in Equation (10) to obtain the new coordinates so as to identify and quantify the actual shape change of a specimen. This can be described with the following Equation (12):

(12) $$\left(x^{\prime },y^{\prime },z^{\prime },1\right)=\;\left[M\right]*\left(x,y,z,1\right),$$

where, $\left(x^{\prime },y^{\prime },z^{\prime },1\right)$ is the point coordinates w.r.t. the new basis; $\;\;\left(x,y,z,1\right)$ is the point coordinates w.r.t. the old basis; $\left[M\right]$ is the transformation matrix.

### 3.3. Estimation of the Deflection Curve from the Loading Scenario

After obtaining the coordinate transformation, it is possible to consider changes in the shape of an object formed by the point cloud that are equivalent to the actual object. In the last phase of the proposed algorithm, we fit the curved surface as a 2nd degree curve on the longitudinal axis and a linear curve on the transverse axis for each individual loading scenario using the transformed point clouds. The GA fitness function appears more complicated than the previous one since the error metric formulation is now considered as a two degree curve caused by loading. This can be described by the following Equation (13):

(13) $$Fitness\;value=\;\sum_{i=0}^{N}{d_{i}}^{2}=\;\sum_{i=0}^{N}{\left(P_{i}-F\left(A,B,C,D,E\right)\right)}^{2}$$

where $d_{i}^{2}$ is the squared error which depicts the difference between the actual data and the model; $P_{i}$ is the measured data of the *z*-coordinate of a point cloud; $F\left(A,B,C,D,E\right)=\left[A{x_{i}}^{2}+Bx_{i}+Cy_{i}+Dx_{i}y_{i}+E\right]$; $x_{i}$ is the measured *x*-coordinate of a point cloud; $y_{i}$ is the measured *y*-coordinate of a point cloud; *A*, *B*, *C*, *D* & *E* are the coefficient parameter of the model; and *N* is the number of points in the point cloud data.

Like the unloading scenario, the resulting point cloud from loading is fitted as a curved surface according to the parameters obtained from genetic optimization. As discussed earlier, the method of finding suitable parameters for the optimization function, i.e., Taguchi’s Design, is also utilized here. Because we assumed that lateral torsional buckling of a specimen is negligible due to the stiffeners, the longitudinal profile deflected shape of the specimen is our main focus. As a result, taking the longitudinal line which lies on the curved surface as a deflection curve makes it easy to estimate the deflection of the required position. Even though we set the local structural coordinates according to the intersection between the upper flange and the front side of the web, the center line of the deflected shape of a curved surface (flange) accurately describes the actual effect of the loading on the specimen. According to our experimental setup, which is a fixed-fixed support of beam, it is known that there is zero deflection at the end point of the specimen. Finally, the results obtained from the actual LVDT measurements and the analysis of what is proposed are compared and contrasted.

## 4. Experimental Study

**Figure 6:**
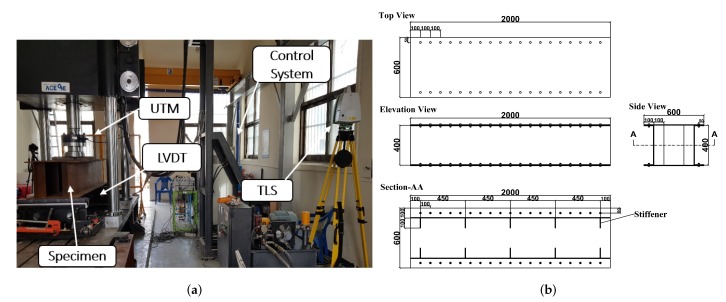
(**a**) Photographic view of the experimental Setup; (**b**) Detailed drawing of the specimen and setup.

The proposed method has been validated through experiment carried out at Sungkyunkwan university. The experiment involved a steel box girder section specimen under different loadings. The experiment took place at the Concrete Material Lab (Suwon), where the University is located. Figure 6 shows how all the entities are synchronized during the experiment. As shown in Figure 6, the main entities that play essential roles during the experiment are:

- Universal Testing Machine (UTM): This device is related mainly to the loading. The load is applied perpendicularly midway from the top flange face with the help of a hydraulic system. The loading system is controlled in real time via the UTM, i.e., the hydraulic power unit, load measuring unit and control devices, which are linked with the loading unit. ACE-USS200 model of Servo-Hydraulic Universal Testing Machines, which having 200 ton loading capacity, was utilized for this study. Servo-Hydraulic UTM can be controlled via a multi-functional remote control handset that is located on the frame, a digital control unit or Material Testing Program (MTP) software was installed on the PC connected to the Control Unit. It can carry out tensile and yield, compression, flexure tests with load and displacement controls.
- Linear variable displacement transducer (LVDT): LVDT is a sensor that converts the linear movement of the object the LVDT is coupled to into a variable corresponding to the electrical signal proportional to that movement. This contact sensor measures the real time displacement of a specimen by attaching the rod element, which is a combination of the core, core extension, and probe tip, lightly to the bottom flange face during unloading. CDP-50 type of LVDT was utilized for our experiment.
- Terrestrial laser scanning(TLS): It measures a scanned object by emitting laser pulses and recording the subsequent intensity of their return after reflection. Leica scan station C5 scanner which is operated based on the time of flight principle was utilized for our experimentation. We have used the highest resolution mode of resolution. According to Leica specifications, this kind of mode has 0.02 m × 0.02 m resolution. Furthermore, there are 2530 × 2181 points in the horizontal and vertical directions, respectively.
- Specimen: The beam utilized for this study is a steel box girder, SS 400-6T, which has dimensions of 0.4 m × 0.8 m × 2 m. the specimen has a transverse stiffener, which stiffens the flange and web against out of plane deformation. The specimen is welded every 45 cm throughout the entire span in both the right and left webs inside the box. As a result, transverse deformation of the specimen is trivial in this study.

### 4.1. Design of the Experiment

**Figure 7:**
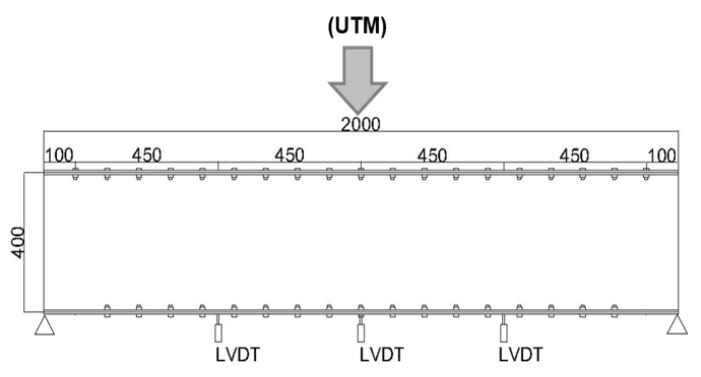
Illustration of LVDT sensor’s position.

The indoor experiment is setup as follows:

- Setting the position of a specimen through the UTM machine keeps all the necessary alignments both horizontally and vertically. The scanner device stands 2.5 m away from the front web face while considering which factors affect the accuracy of the data. Target color, incidence angle, range and intensity are the main factors that affect the point cloud noise and parametric model fitting [13]. Consequently, this setup considers all the results from Bolkas et al. in accordance with the available space in the laboratory.
- The contact sensor (LVDT) is mounted below the specimen at three different positions. One is at the center and the other two LVDTs are fixed 55 cm from the left and right edges individually, as shown in Figure 7. These LVDTs and the UTM machine are connected to a computer.
- The specimen is scanned without any loading by setting the required field of view. The field of view for scanning an object should reduce the outliers caused by objects out side of the target.
- Once we are done scanning the specimen scene without loading, we apply loading via the UTM until the center LVDT reading reaches 1mm. The 1 mm sag is attained at a 57.33 KN loading. Again, the scanning process starts over by pausing the applied load and keeping the 1 mm sag.
- In this fashion, specimen scanning is carried out for different deflection sizes for the corresponding loadings. Table 2 summarizes the induced load for each case along with their corresponding LVDT sensor readings.
- After capturing all the necessary data with the USB, which was plugged in to the scanner during the scanning process, we changed the file format from .PLY to .PTS using cyclone which is a software module of Leica, for the purpose of using the cloud compare software. Once we have the data file format which is capable to utilize via with cloud compare, it is easy to apply manual segmentation of an object entity, removing the outliers and preparing the data for further analysis. one of the advantage of this software regarding with removal of an outliers, it provides segmentation command in different shape using polylines. Figure 2a,b, which depict the data with and with out the outliers respectively, are obtained from this software.

### 4.2. Validation Results

As described in previous sections, the specimen is scanned exactly after excitation, which leads to changes in shape followed by scanning without any loading. Once all specimen shape information is obtained for each load case, removal of the outliers and segmentation are carried out. The point cloud obtained from the experiments was processed using the proposed algorithm. Figure 2b depicts the resulting cloud data after preprocessing.

#### 4.2.1. Selection of Optimal Parameters for GA

**Figure 8:**
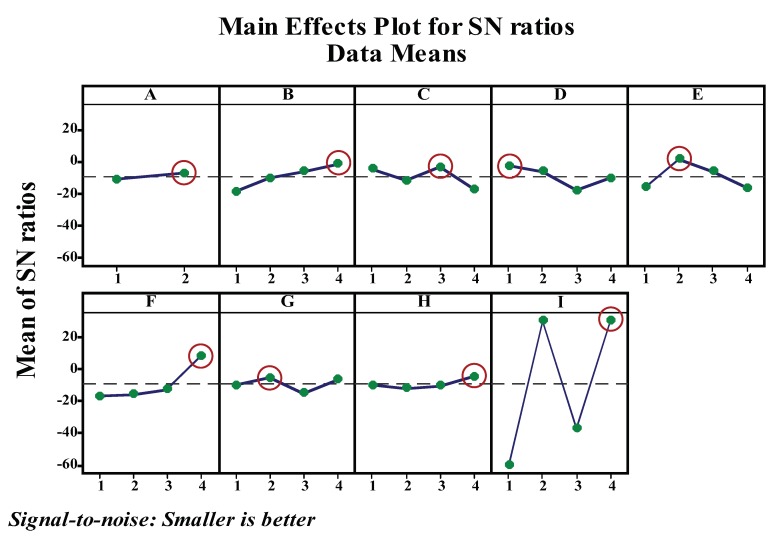
Signal to noise ratio plot for each of the GA parameters.

As shown in Table 1, eight control parameters at four values and one with two are identified in this study. Due to the number of parameters and their values considered in here, $L32^{\prime }\left(2^{1}x4^{8}\right)$ orthogonal array is suitable for the experiment design. Orthogonal Arrays (OAs) provide a set of well balanced and minimal experiments. This array assumes that there is no interaction between any two factors. The experiments are repeated five times to increase the consistency of the experiment response. The analysis for the proposed Taguchi design is analyzed using a statistical software MINITAB19. Signal-to-noise ratios (SNR), which are log functions of the desired output as described in Equation (5), serve as objective functions for optimization, as well as help in data analysis and the prediction of optimum results. In the Taguchi method, the word “optimization” means determining the best values for the control factors. In turn, the best values for the control factors are those that maximize the signal-to-noise ratios. This can also be described as the resulting best values for the control factors so that the fitness functions are negligible, since the fitness functions are directly proportional to the error metric during plane fitting. Based on Taguchi two-step method rules, performance of a design is checked by maximizing the SN ratio and adjusting the mean to the target values. The Higher values of the Signal-to-noise ratio identify control factor settings that minimize the effects of the noise factor. In this study, the fitness value which is a response for the design experiment has a smaller the better characteristic. Figure 8 depicts the graphical representation of a robust level of parameter for the Genetic Algorithm. As a result, the optimal combination of control factors for the genetic algorithm to minimize the error is depicted in Table 3.

#### 4.2.2. Computation of Deflection Based on the Genetic Algorithm

Because of the nature of a lidar system, which is a collection of points, it is difficult to point out the edge of a specimen that would be considered as the “x-axis” of the local structural coordinate system. Therefore, representing the point cloud data by definable mathematical elements, planes, and lying one axis of a coordinate on a line which is obtained by correlating these planes are the easiest and optimal approach. A stochastic optimization, the genetic algorithm in this case, is utlized in this study to find the optimal planes that are most compatible with the data acquired. Even though this kind of optimization is prominently used for nonlinear forms and forms for which no derivative information exists [39], it also plays an enormous role in finding the optimal solution even in heavily noisy spaces [40]. Accordingly, the fitness function for the genetic algorithm is directly derived from the sum of the squares of the residuals, as shown in Equation (3).

**Figure 9:**
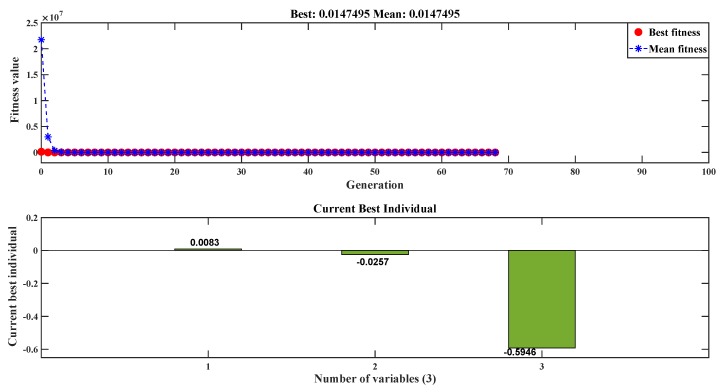
Genetic Algorithm generation and individuals value for unloading case.

Using the optimal control parameters shown in Table 3, the individual plane entity was fitted using the fitness function for the GA from the least square error (Equation (3)), as shown in Figure 5. The coefficients of the plane equation, i.e., the best individuals in genetic algorithm language, are obtained through a built-in MATLAB code, the Global Optimization toolbox, after determining the optimal parameter. By utilizing the Taguchi experimental design and obtaining the optimal parameter, the fitness values started to converge in the early generations (around the 3rd) and stopped at the 68th generation by satisfying the 1st stopping criteria (i.e., the average change in fitness value is less than the function tolerance). Figure 9 depicts the mean and best fitness values with respect to each generation and the best individual values for the flange entity plane.These best individuals are 0.0083, −0.0257 and −0.5946. Therefore, the resulting planes which represent the acquired data for each entity of the specimen looks as follows:

$G\left(I,J\right)=\;-0.5946+0.0083\cdot I-0.0257\cdot J$… Equation of a plane that represents the flange part;
$F\left(C,D\right)=\;-7.491-2.0996\cdot C+0.0123\cdot D$ … Equation of a plane that represents the web part.

After obtaining the plane equation that represents the web part in a similar way to the flange part, the 3D intersection line $L_{i}$, which is defined as the longitudinal axis of the local beam structural coordinates, is developed by considering Equation (7) parametrically. This gives us:

$$\begin{array}{c} x=1.0003\cdot t;\\ y=-7.493-2.0987\cdot t;\\ z=-0.4022+0.0621\cdot t. \end{array}$$

**Figure 10:**
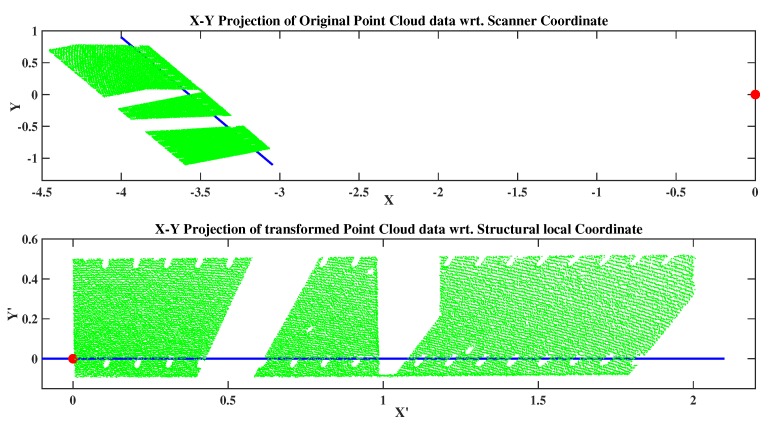
Flange point cloud before and after transformation of the coordinates.

Now, it is possible to position at least one axis of the new coordinate system, the x-axis in our case, along the intersection line described in the above figure. From Figure 5, the difference between points $O^{\prime }$ and Q gives us the vector which lies on the new x-axis, called *X*${}^{\prime }$. Before performing the rotation, we should determine the geometric correlation between the vector $O^{\prime }$Q and the old basis (*X*,*Y*,*Z*) after translating of point *O* to point $O^{\prime }$. Figure 10 shows the resulting point cloud data after performing the required transformation. This information is helpful in quantifying the deflection of a structure by applying the transformation matrix to the acquired point cloud data during the loading scenario.

**Figure 11:**
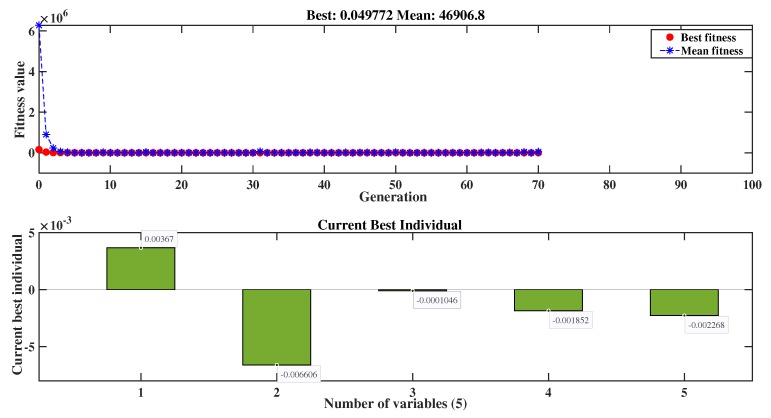
Genetic algorithm generation and individual values for a deflected shape.

**Figure 12:**
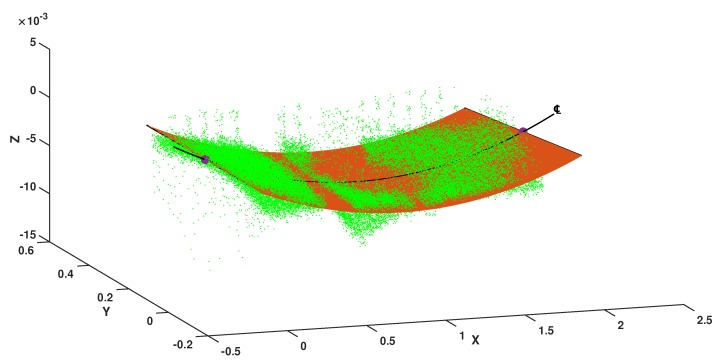
Deflected shape of flange entity with deflection curve at the enter line.

**Figure 13:**
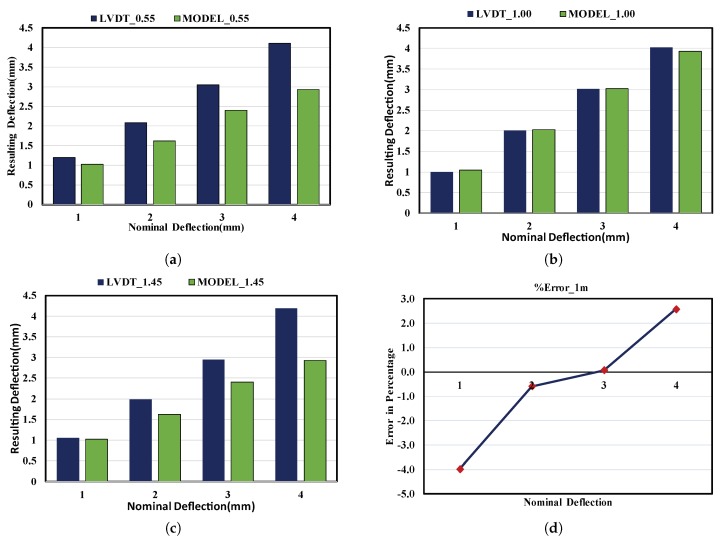
(**a**) model vs LVDT @ 0.55 m;(**b**) model vs LVDT @ 1.00 m; (**c**) model vs LVDT @ 1.45 m; (**d**) Absolute Error for model at mid-span.

Similar to the loading scenario, the fitness function for the loading case is obtained using Equation (13). A 2nd degree of polynomial curve is constructed for the fitness function considering the complexity degree of the structure based on given experimental study. This leads us to compute the best individuals similar as before, but here, we have five individual parameters for the curved surface, as illustrated in Figure 11. This provides the deflection shape of the top flange specimen as depicted in Figure 12. Even though our x-axis lies on the intersection between the flange and web as shown in Figure 5, the center line in Figure 12 indicates a larger deflection shape of the structure induced by the point load. Therefore, squeezing the plane towards the center transversely from each side gives us the required hypothetical deflection curve, which enables us to calculate the deflection of a specimen throughout its entire length. Here, only the deflection curve, which appears 4 mm at the center LVDT sensor, is shown diagrammatically. The rest of the deflections constructed using the proposed model along with their corresponding load contact sensor measurements are depicted in Figure 13.

The resulting equation of center line ℄ from Figure 12 was calibrated by considering its 2nd degree curve behaviour. Some boundary conditions appear because of the support condition. As shown in Figure 6a, the support conditions used in this experiment lead us to determine the displacement and the moments at the support (i.e., $\delta_{\left(x=0,x=L\right)}=0\;\;\&\;\;M_{\left(x=0,x=L\right)}=0$), such that the moment at the midpoint is a maximum.This calibration is needed because of the inherent random error of the instrument and the natural difference between the theoretical and experimental solutions. In the following, we illustrate how a signal equation can be calibrated by employing the boundary equation for the 4 mm loading scenario. The deflection curve obtained from the proposed analysis is as follows:

$Z=-2.138\times 10^{-3}-7.092\times 10^{-3}\cdot X+3.925\times 10^{-3}\cdot X^{2}$

According to basic beam theory, the max deflection for a simply supported beam must attained at the middle of the span. This implies that

*dZ*/*dx* = 0,
$dZ/dx=7.85\times 10^{-3}\cdot x-7.092\times 10^{-3\;}=0$

x=0.90344…… while, *x* must be 1. This tells us the resulting signal deviates by (1−0.90344)=0.09656 from the real value. Therefore, the signal needs to be adjusted by +0.09656 along the x-direction and −0.002138 along the z-direction.

$dZ/dx=7.85\times 10^{-3}\cdot -K=0\Leftrightarrow x=1\;\;\;∴\;\;\;K=7.85\times 10^{-3}$
$dZ/dx=7.85\times 10^{-3}\cdot x-7.85\times 10^{-3}$
$\int dZ/dx=\;\int \left(7.85\times 10^{-3}\cdot x-\;7.85\times 10^{-3}\right)$
$Z=3.925\times 10^{-3}\cdot X^{2}-7.85\times 10^{-3}\cdot X$

Thus, the deflection curve after calibration of the measured data is obtained for the loading scenario *z* = 4 mm.

Finally, the deflection of a loaded beam was estimated from the deflection curve and validated via the corresponding contact sensor (LVDT). The difference between the LVDT and proposed methodology results are illustrated in Figure 13.The model for the center LVDT, which is at 1 m, almost coincides with the actual sensor measurements, while the models for the edge LVDTs are not. This is happened because;

- Since the specimen has transverse stiffener at 45 cm apart along both sides, it is very stiff along with the loading. However, the stiffness of a specimen is not uniform throughout the span with increasing load. A part of the specimen in between two stiffeners has not been equally disturbed with the part where exactly stiffener is welded. Consequently, the global deflection curve may not be expected exactly as a 2nd degree parabolic.
- Secondly, the nature of a specimen has also its part in affecting the result for the edge LVDTs. Hence, the beam is labelled as a deep beam because of its span-to-depth ratio and the concentrated load with it. Therefore, the shear effect is predominant than flexural in our specimen. This implies that the deflection curve which is expected from the flexural effect is affected to some extent. Even the data shown for edge LVDTs are biased by this effect.

As a result, the proposed approach of representing the deflection shape in terms of a parabolic curve may only accurately capture the deflection formed throughout the whole span length except for around the mid-span. For emphasis, in the case of edge LVDTs in Figure 13a,c, the resulting deflection from the LVDT in each load scenario is almost the same as with the nominal one. However, this situation is incompatible with the proposed model since we are dealing with a deflection curve considered as a 2nd degree polynomial curve. It is obvious that if we consider a deflection curve as explained above while keeping the max deflection (at the center) equal to the nominal deflection, the deflection at the edge LVDT is less than the nominal one. Consequently, the proposed methodology is validated only for the middle LVDT sensor. As shown in Figure 13d, the proposed algorithm is accurate and effective for nominal deflections of 2 and 3 mm since the error is between ±1. For the case of nominal deflections of 1 and 4 mm, the error increases to ±4 and ±2.6, respectively.

## 5. Conclusions

In this work, we proposed a method for estimating the deflection of a beam based on a non-contact sensor, TLS. We simultaneously applied regression curve fitting with a genetic algorithm. The accustomed method, least squares regression fitting, is used to determine the fitness function which measures the suitability of the solution generated by the GA. The GA minimizes the fitness function obtained from the error equation. In addition, transformation of the coordinates is a crucial step in this study. This is because in order to define any geometrical changes in a structure with respect to its own axes, the scan data obtained from the device must also visualize the target structure with all its parts and elements so that periodic maintenance is predetermined. The equation for the curve is extracted from the surface fitting after transforming the coordinates of the data. This equation represents the deflection curve and, therefore, allows us to estimate the deflection value of a beam at any position across its span. The deflection value at various points was validated using the corresponding direct contact sensor, LVDT.

The proposed method is practical in applying the concept of structural health monitoring in different structures using non-contact sensors. Besides computation of a deflection, the geometric shape of a structure at the time of scanning can be visualized throughout this proposed methodology. This leads to regulating a structure health condition all over the area. As shown in the result, this algorithm is suitable for long-spanned beam. However, it is also effective in determining the deflection around the mid-span.

## Figures and Tables

**Table 1:** Genetic Algorithm Parameters and their levels.

Factors	Levels				
Code	GA Parameter	1/Default	2	3	4
A	Migration Direction	Forward	Both	-	-
B	Population Size	50	100	150	200
C	Fitness Scaling Function	Rank	Proportional	Top	Shift Linear
D	Selection Function	Stochastic Uniform	Remainder	Roulette	Tournament
E	Elite Count	2	5	10	20
F	Crossover Function	Scattered	Two Point	Heristic	Arthmetic
G	Crossover Fraction	0.8	0.6	0.4	0.2
H	Mutation Function	Gaussian	Uniform	Adaptive Feasible	Constraint Dependent
I	Hybrid Function	None	FminSearch	Patternsearch	Fminunc

**Table 2:** Load cases and LVDT readings for the corresponding nominal deflection.

Loading	Nominal	Load	LVDT Reading (mm)		
	Deflection	(KN)	0.55 m	1.00 m	1.45 mm
Case 1	1mm	57.33	1.204	1.005	1.055
Case 2	2mm	200.47	2.086	2.014	1.99
Case 3	3mm	380.85	3.046	3.022	2.949
Case 4	4mm	480.84	4.109	4.029	4.188

**Table 3:** Optimal combination parameters from the response table.

		A	B	C	D	E	F	G	H	I
Rank	SN Ratio	9	4	6	5	3	2	7	8	1
	Mean	9	4	5	6	8	2	7	3	1
Optimum Level	SN Ratio	2	4	3	1	2	4	2	4	4
	Mean	1	1	4	3	4	3	2	2	1
Combination		A2	B4	C3	D1	E2	F4	G2	H4	I4

## Abbreviations

The following abbreviations are used in this manuscript:

LIDAR	Light Detection And Ranging
TLS	Terrestrial Laser Scanning
GA	Genetic Algorithm
3D	Three-Dimesion
LVDT	Linear Variable Differential Transformer
LS-GA	Least Square Based Genetic Algorithm
DOE	Design of Experiemnt
RGB	Red Green Blue
SNR	Signal to Noise Ratio
UTM	Universal Testing Machine
OA	Orthogonal Arrays

## References

[1] Chong K.P., Zhu S.Y. (2018). Innovative technologies in manufacturing, mechanics and smart civil infrastructure. Int. J.Smart Nano Mat..

[2] Maskuriy R., Selamat A., Ali K.N., Maresova P., Krejcar O. (2019). Industry 4.0 for the construction industry—How ready is the industry?. Appl. Sci..

[3] Giurgiutiu V., Cuc A. (2005). Embedded non-destructive evaluation for structural health monitoring, damage detection, and failure prevention. Shock Vibr. Digest.

[4] Farrar C.R., Keith W. (2007). An introduction to structural health monitoring. Philos. Trans. R. Soc. Math. Phys. Eng. Sci..

[5] Achenbach J.D. (2000). Quantitative nondestructive evaluation. Int. J. Solids Struct..

[6] Chan T.H., Yu L., Tam H.Y., Ni Y.Q., Liu S.Y., Chung W.H., Cheng L.K. (2006). Fiber bragg grating sensors for structural health monitoring of tsing Ma bridge: Background and experimental observation. Eng. Struct..

[7] Behnia A., Chai H.K., Shiotani T. (2014). Advanced structural health monitoring of concrete structures with the aid of acoustic emission. Constr. Build. Mater..

[8] López-Higuera J.M., Cobo L.R., Incera A.Q., Cobo A. (2011). Fiber optic sensors in structural health monitoring. J. Light. Technol..

[9] Terroba F., Frövel M., Atienza R. (2019). Structural health and usage monitoring of an unmanned turbojet target drone. Struct. Health Monit..

[10] Giurgiutiu V. (2005). Tuned Lamb wave excitation and detection with piezoelectric wafer active sensors for structural health monitoring. J. Intell. Mater. Syst. Struct..

[11] Cha G., Park S., Oh T. (2019). A terrestrial LiDAR-based detection of shape deformation for maintenance of bridge structures. J. Constr. Eng. Manag..

[12] Soudarissanane S., Lindenbergh R., Menenti M., Teunissen P. (2011). Scanning geometry: Influencing factor on the quality of terrestrial laser scanning points. Isprs J. Photogramm. Remote. Sens..

[13] Bolkas D., Martinez A. (2018). Effect of target color and scanning geometry on terrestrial LiDAR point-cloud noise and plane fitting. J. Appl. Geod..

[14] Cabaleiro M., Lindenbergh R., Gard W.F., Arias P., Van de Kuilen J.W.G. (2017). Algorithm for automatic detection and analysis of cracks in timber beams from LiDAR data. Constr. Build. Mater..

[15] Kim K., Kim J. (2015). Dynamic displacement measurement of a vibratory object using a terrestrial laser scanner. Meas. Sci. Technol..

[16] Xu X., Yang H., Neumann I. (2015). Concrete crack measurement and analysis based on terrestrial laser scanning technology. Sens. Transducers.

[17] Kitratporn N., Takeuchi W., Matsumoto K., Nagai K. (2018). Structure deformation measurement with terrestrial laser scanner at pathein bridge in myanmar. J. Disaster Res..

[18] Yang H., Omidalizarandi M., Xu X., Neumann I. (2017). Terrestrial laser scanning technology for deformation monitoring and surface modeling of arch structures. Compos. Struct..

[19] Lam S.Y. (2006). Application of terrestrial laser scanning methodology in geometric tolerances analysis of tunnel structures. Tunn. Undergr. Space Technol..

[20] Park H.S., Lee H.M., Adeli H., Lee I. (2007). A new approach for health monitoring of structures: Terrestrial laser scanning. Comput. Aided Civ. Infrastruct. Eng..

[21] Cabaleiro M., Riveiro B., Arias P., Caamaño J.C. (2015). Algorithm for beam deformation modeling from LiDAR data. Measurement.

[22] Gordon S.J., Lichti D., Stewart M., Franke J. Structural Deformation Measurement Using Terrestrial Laser Scanners. Proceedings of the 11th FIG Symposium on Deformation Measurements.

[23] Zogg H.-M., Ingensand H. (2008). Terrestrial laser scanning for deformation monitoring: Load tests on the Felsenau Viaduct (CH). Int. Arch. Photogramm. Remote. Sens. Spat. Inf. Sci..

[24] Olsen M.J., Kuester F., Chang B.J., Hutchinson T.C. (2010). Terrestrial laser scanning-based structural damage assessment. J. Comput. Civ. Eng..

[25] Cabaleiro M., Riveiro B., Arias P., Caamaño J.C. (2016). Algorithm for the analysis of deformations and stresses due to torsion in a metal beam from LIDAR data. Struct. Control. Health Monit..

[26] Li H., Chang J., Xu F., Liu Z., Yang Z., Zhang L., Zhang S., Mao R., Dou X., Liu B. (2019). Efficient lidar signal denoising algorithm using variational mode decomposition combined with a whale optimization algorithm. Remote. Sens..

[27] Liu X., Zhang Z., Peterson J., Chandra S. The Effect of LiDAR Data Density on DEM Accuracy. Proceedings of the International Congress on Modelling and Simulation (MODSIM07).

[28] Mao J. (2012). Noise reduction for lidar returns using local threshold wavelet analysis. Opt. Quantum Electron..

[29] Fang H.T., Huang D.S. (2004). Noise reduction in lidar signal based on discrete wavelet transform. Opt. Commun..

[30] Salgado C.M., Azevedo C., Proença H., Vieira S.M. (2016). Noise versus outliers. Secondary Analysis of Electronic Health Records.

[31] Jung B.K., Cho J.R., Jeong W.B. (2015). Sensor placement optimization for structural modal identification of flexible structures using genetic algorithm. J. Mech. Sci. Technol..

[32] Kumar R., Ramachandra L.S., Roy D. (2004). Techniques based on genetic algorithms for large deflection analysis of beams. Sadhana.

[33] Almongy H.M., Almetwaly E.M. Comparison between Methods of Robust Estimation to Reduce the Effect of Outliers. https://www.researchgate.net/publication/326557510.

[34] Gibson C.G. (2003). Elementary Euclidean geometry: An Introduction.

[35] Yan X., Su X. (2009). Linear Regression Analysis: Theory and Computing.

[36] Holland J.H. (1992). Genetic algorithms. Sci. Am..

[37] Goldberg D.E. (1989). Genetic algorithms in search, optimization, and machine learning. Choice Rev. Online.

[38] Gulsen M., Smith A.E., Tate D.M. (1995). A genetic algorithm approach to curve fitting. Int. J. Prod. Res..

[39] Karr C.L., Stanley D.A., Scheiner B.J. (1991). Genetic Algorithm Applied to Least Squares Curve Fitting.

[40] Messa K., Lybanon M. Curve Fitting Using Genetic Algorithms. https://apps.dtic.mil/dtic/tr/fulltext/u2/a247206.pdf.

[41] Shrestha A., Mahmood A. (2016). Improving genetic algorithm with fine-tuned crossover and scaled architecture. J. Math..

[42] Forouraghi B. (2000). A genetic algorithm for multiobjective robust design. Appl. Intell..

[43] Majumdar A., Debashis G. (2015). Genetic algorithm parameter optimization using Taguchi robust design for multi-response optimization of experimental and historical data. Int. J. Comput. Appl..

[44] Taguchi G., Chowdhury S., Wu Y. (2005). Quality engineering: The taguchi method. Taguchi’S Qual. Eng. Handb..

[45] Dehnad K. (2012). Quality Control, Robust Design, and the Taguchi Method.

[46] Galarza A.I.R., Seade J. (2007). Introduction to Classical Geometries.

[47] Anton H., Chris R. (2013). Elementary Linear Algebra, Binder Ready Version: Applications Version.

[48] Agoston M.K., Max K.A. (2005). Computer Graphics and Geometric Modeling.

